# Round the Hospitals

**Published:** 1917-06-09

**Authors:** 


### ROUND THE HOSPITALS.

Matrons, Sisters, and Nurses will recognise the supreme importance of the decision embodied in the resolutions we publish this week of the Association for the Promotion of State Registration of Nurses in Scotland, which embraces, we learn, an overwhelming majority of trained Scottish Nurses. The wording of the resolutions indicates that the Central Committee by its censured action has placed itself legally in a position which a court of law would almost certainly rule was destructive of its authority to speak in a representative capacity for the nurses attached to the eight societies which form the Central Committee, and disqualifies it from all claim whatever to speak or act or represent the trained nurses of the United Kingdom. These legal aspects may have a great importance later on when the Registration question comes again before Parliament, and a mandamus may, and possibly will, dispose of the Central Committee's Bill, which has not and cannot legally claim to have behind

**ROUND THE HOSPITALS—(*continued*).**

it the eight societies out of which it was formed. The members of these associations have never been asked nor have they yet approved the policy of "Divide" and Destruction so dear to the few active spirits on that Committee, who have taken the bit in their mouths and defied the Association members, ignoring the fact that without their support they have neither authority nor status. British Nurses are not the fools that they are apparently thought to be by the few individuals who have destroyed, in the case of the Central Committee, that representative government of which they are always talking, but for which by their actions they have continuously shown the supremest contempt. We hope matrons, sisters, and hospital authorities, as well as every intelligent trained nurse, will grasp these points and make it their business to bring them forcibly to the notice of every trained nurse with whom individually they may have touch. Meanwhile English, Welsh, and Irish nurses owe grateful thanks to the Scottish Association for the Promotion of State Registration for the stand they have made, and the wise policy they have pursued in exposing the instability of the Central Committee's position *vis-à-vis* with Parliament, British courts of law, and British Nurses throughout the United Kingdom and its Dominions. Trained British Nurses are solid for the rule *not to sign their names to any petition*, and to withdraw their names if inadvertently given.

At a meeting of the Executive Committee and members of the Association for the Promotion of the State Registration of Nurses in Scotland (one of the largest of the eight societies which form the Central Committee), held in the Garlstion Hall, George Street, Edinburgh, on Thursday, May 31, the following resolution was proposed by Professor Glaister, seconded by Miss Gill, and unanimously agreed to:—"The following resolution passed at the meeting held on November 24, 1916, *was re-affirmed*: 'That the Association for the Promotion of the Registration of Nurses in Scotland protests against the action of the Central Committee for the State Registration of Nurses in London, in determining (1) to break off negotiations with the College of Nursing, Ltd., at a time when unanimity of action seemed so hopeful, and (2) to proceed with its own Bill without having first consulted the members of the Associations represented on the Central Committee in view of the fact that substantial progress in the negotiations between the two bodies towards an agreed Bill seems to have been achieved,' adding the following, 'The Executive Committee strongly recommends members of the Association for the Promotion of the Registration of Nurses in Scotland not to sign any petition to the Prime Minister such as is at present circulated by the Society for the State Registration of Trained Nurses, 431 Oxford Street, London.'" The Right Hon. Lord Inverclyde, President of the Association, was unavoidably prevented from attending the meeting. Professor Glaister occupied the chair, and among others present were Colonel D. J. Mackintosh, C.B., M.V.O., Dr. Maxton Thom, Sir James Affleck, M.D., Dr. Claude Ker, Miss Logan, Miss Turnbull, Miss Thyme, Miss Graham, and Miss Pabla Sterling.

A very successful meeting on behalf of the College of Nursing was held on Saturday, June 2, at the Women's Union Rooms of the University of Manchester. The meeting was called by Miss Smith, of the Withington Hospitals, who is now the representative of the Poor-Law Infirmary Matrons' Association in the Manchester area, with the object of meeting the superintendent nurses and of asking the matrons of all Poor-Law hospitals to meet the superintendent nurses and their staffs. About 150 certificated nurses were present, and a pleasant and interesting afternoon was spent.

Miss Barton, President of the Poor-Law Infirmary Matrons' Association, gave an account of the Association and its work. On behalf of the Association she welcomed the superintendent nurses, who have lately been invited to join as associates. Miss Rundle, secretary of the College of Nursing, then gave a full account of the origin, objects, and aims of the College. She appealed to those present to awake to their responsibilities and to send her any suggestions for improvements and for extending the usefulness of the College. Miss Girdlestone, late matron of the Crumpsall Infirmary, who was very heartily welcomed by her numerous old friends, urged the claims and advantages of the College and declared that the nurses did trust their matrons, whom they wanted to plan for them, knowing they would do their very best. Miss Gibson, late matron of the Birmingham Infirmary, advised all nurses to keep their aims and ideals high—"Put your star right up in front of you and try to follow it." She urged all nurses to read both sides of the questions now under debate. Miss Burgess, matron of Crumpsall Infirmary, and Miss Ross, matron of the Hope Hospital, proposed votes of thanks. Many of the audience, after enjoying Miss Smith's hospitality, were taken round the Royal Infirmary by Miss Sparshott.

Birmingham Guardians have been considering the salaries paid to nurses up and down the country in connection with the great difficulty now experienced in attracting candidates for the higher posts of ward sister and others, and also suitable probationers. Higher salaries are being offered in military and other hospitals, whilst the inducement of war bonuses in many places is being held out. The Birmingham Guardians are strongly of opinion that it is essential, in order to retain the services of the existing officers and to obviate difficulties in filling vacancies, that special increases should be made, and they are therefore asking the Local Government Board to sanction special temporary increases of salary, to cease three months after the declaration of peace, to the undermentioned classes of nurses:—£10 per annum to first and second assistant matrons, first and second night sisters, home sisters, maternity sisters, female

For "Food in War Time," see p. 194; "Mothercraft and Child Welfare," p. 195; "Matrons' and Sisters' Appointments," p. iv.

epileptic block sister, infectious block sister, theatre sisters, and charge nurses, and £5 per annum to probationers and staff nurses.

---

AT the recent annual meeting of the Purey Cust Nursing Home, York, presided over by the new Dean of York, special mention was made of the honour conferred upon the lady superintendent, Miss Morgan, through the award to her by the King of the Royal Red Cross, First Class. Despite the difficulties due to the depletion of the staff of the Home, owing to the demands of the war, the lady superintendent being one of the many members of the staff whose services were thus claimed, the Home has carried on successfully and its work has been much appreciated, as is shown by the fact that during the year 649 cases were treated in the Home, and 15,424 visits were paid by the district nurses, totals which compare favourably with past years.

---

WE have long contended that institution workers were entitled to enjoy a general level of comfort in their meals not inferior to that which prevails in home circles. At the time when margarine first began to appear in the shops it had little but cheapness and wholesomeness to recommend it. Its food value for the price was admittedly high, but it did not please. We considered it unfit for institution use, and said so. Recent advances in the processes of manufacture have now established beyond question that a valuable and palatable addition has been made to our food resources in the preparation of this substance from cocoanut oil, since it has been successfully freed from the somewhat rank odour and flavour which clung to the original product. And now while all the world is expressing its sense of the fact that we are at war by introducing margarine on the table as well as in the kitchen, there are still institution managers who go on paying 2s. a lb. and even more for a scanty supply of butter, under the impression that they would inflict hardship on staff and inmates alike if they substituted for it the margarine which is now placidly consumed in almost every private household in the country. The great lady who entertained her friends at a sumptuous tea a short time ago and revealed later to her admiring guests that every cake and every morsel of bread and "butter" had been compounded of margarine took the best possible way of rubbing off prejudice. We commend the plan to housekeepers who still hold aloof from this necessary and harmless economy. But care is needed to ensure that a high quality of margarine is always purchased.

---

WE have received the following note of appreciation from Miss M. Rose, the present hon. lady superintendent of the Children's Hospital for Hip Disease, Sevenoaks:—

I feel I cannot delay longer to thank you very heartily for your most sympathetic notices of Miss Emily Jackson in your columns. It seemed to me when you wrote after her decease that you must have known her personally, for there never was a truer record of her than that voiced by you. "She did not court the limelight." Who should know better than her friend and co-worker of thirty-five years?

